# The Role of Phase-Change Memory in Edge Computing and Analog In-Memory Computing: An Overview of Recent Research Contributions and Future Challenges

**DOI:** 10.3390/s25123618

**Published:** 2025-06-09

**Authors:** Alessio Antolini, Francesco Zavalloni, Andrea Lico, Said Quqa, Lorenzo Greco, Mauro Mangia, Fabio Pareschi, Marco Pasotti, Eleonora Franchi Scarselli

**Affiliations:** 1 Advanced Research Center on Electronic Systems “Ercole de Castro”, Dipartimento di Ingegneria Elettrica e dell’Informazione “Guglielmo Marconi”, University of Bologna, 40123 Bologna, Italy; francesco.zavalloni2@unibo.it (F.Z.); andrea.lico2@unibo.it (A.L.); lorenzo.greco14@unibo.it (L.G.); mauro.mangia@unibo.it (M.M.); eleonora.franchi@unibo.it (E.F.S.); 2 Dipartimento di Ingegneria Civile, Ambientale e dei Materiali, University of Bologna, 40123 Bologna, Italy; said.quqa2@unibo.it; 3 Dipartimento di Elettronica e Telecomunicazioni, Politecnico di Torino, 10129 Torino, Italy; fabio.pareschi@polito.it; 4 STMicroelectronics, 20864 Agrate Brianza, Italy; marco.pasotti@st.com

**Keywords:** phase-change memory (PCM), analog in-memory computing (AIMC), edge computing, neural networks, artificial intelligence, structural health monitoring (SHM), human body monitoring

## Abstract

Phase Change Memory (PCM) has emerged as a promising non-volatile memory technology with significant applications in both edge computing and analog in-memory computing. This paper synthesizes recent research contributions on the use of PCM for smart sensing, structural health monitoring, neural network acceleration, and binary pattern matching. By examining key advancements, challenges, and potential future developments, this work provides a comprehensive state-of-the-art overview of PCM in these domains, highlighting the possible employments of PCM technology in further edge computing scenarios including medical and human body monitoring.

## 1. Introduction

**Figure 1:**
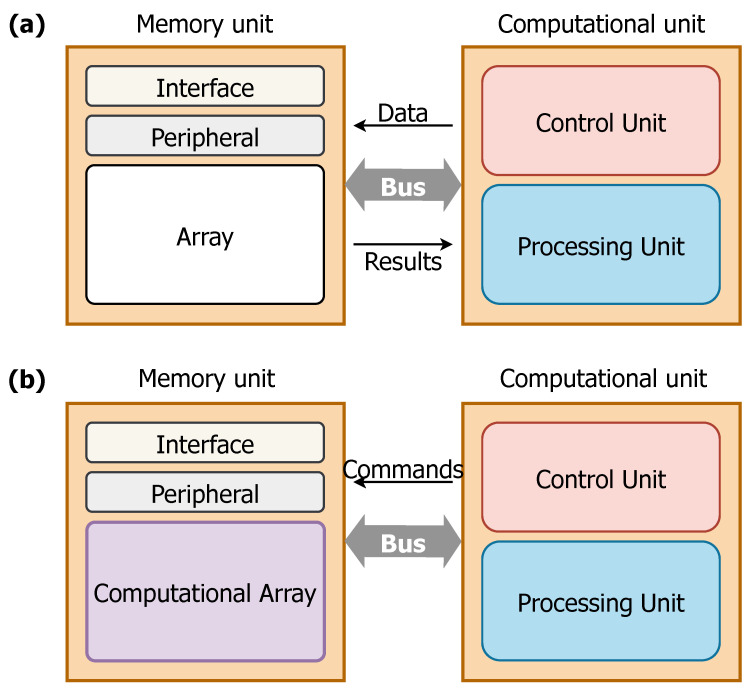
Difference between computations in a conventional architecture (**a**) and the IMC paradigm (**b**).

In-Memory Computing (IMC) is an alternative design approach in the System-on-Chip (SoC) field, where certain computational tasks are performed within the memory unit itself. Figure 1 schematically illustrates the differences in the computation mechanism between a conventional computing system (top) and an in-memory computing one (bottom) [1]. In the first case, the processing unit and memory are physically separated, meaning that data must be constantly conveyed between the two units. This results in significant additional costs in terms of latency and energy [2]. In the case of in-memory calculation, the entire operation is performed within the computational memory unit, avoiding the need to move data into the processing unit. Computations are carried out using the memory matrix and its peripheral circuits without accessing the contents of individual memory elements. This method typically leverages the physical characteristics of memory devices along with their array-level organization, peripheral circuitry, and control logic.

The advantages related to power consumption in IMC architectures arise mostly from the massive parallelism afforded by a dense array of millions of memory devices performing computation. It is also likely that computational time complexity can be further reduced by introducing physical coupling between the memory devices [3], which is an attractive aspect for applications that require simple but repeated operations such as matrix operations or convolutions performed on a large set of data. By blurring the boundary between the processing unit and memory unit, it is possible to achieve significant improvements in computational efficiency; however, this comes at the expense of the generality offered by the conventional approach in which the memory and processing units are functionally distinct from each other. In fact, unlike generic processors, which can perform any type of calculation, IMC supports only a limited set of operations [4].

Analog In-Memory Computing (AIMC) [5] specifically implements the functions required by the IMC paradigm, exploiting analog quantities such as current, voltage, and electrical charge to perform the most common and basic mathematical operations executed in processing units (i.e., sums, accumulations, and multiplications). This implementation is strongly related to the memory support allowing for the execution and the storage of such operations. Among these, Phase-Change Memory (PCM) has emerged as an enabling technology thanks to its high-density and non-volatile storage capability. Thus, massive research effort has focused on developing and demonstrating the potential of PCM for this novel computing approach.

This paper provides a comprehensive overview of the role of Phase-Change Memory (PCM) in enhancing the efficiency and performance of AIMC systems, highlighting its advantages in computational efficiency and power consumption. In addition, this review explores the challenges associated with PCM usage, such as conductance drift and programming variability, and discusses strategies for their mitigation. Furthermore, we examine the practical applications of PCM-based AIMC systems in edge computing, assessing their benefits and limitations compared to traditional architectures. Finally, we consider the potential of PCM technology for future advancements in human body monitoring and other emerging edge computing applications that emphasize its promise for resource-constrained environments. The rest of this paper is organized as follows: in Section 2, the AIMC paradigm and its relation to PCM technology is discussed in detail; Section 3 presents and compares three state-of-the-art AIMC prototypes based on PCM; Section 4, Section 5, Section 6 and Section 7 discuss the employment of such architectures in edge computing scenarios; Section 8 briefly discusses the potential applications of PCM technology in human body monitoring; finally, Section 9 concludes the paper.

## 2. Analog In-Memory Computing

**Figure 2:**
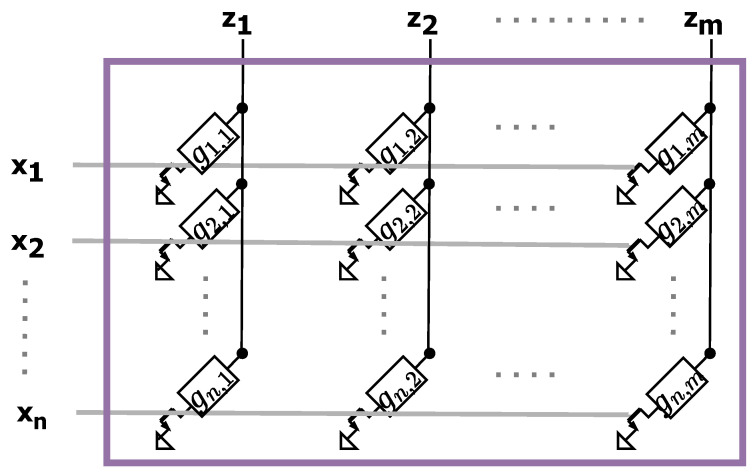
Example of a physical array to perform MVMs for AIMC.

Typical tasks performed in AIMC systems consist of Matrix Vector Multiplication (MVM) functions. In digital systems, these operations are typically simplified to floating-point or fixed-point calculations; instead, as depicted in Figure 2, AIMC for matrix operations exploits the possibility of mapping a 2D matrix into a physical array with an appropriate number of rows and columns in accordance with the mathematical operand. At the intersection between each row and column, a memory element with conductance $g_{i,j}$ represents a generic element of the matrix G involved in the computation. The components of a voltage input vector $x=x_{i=1,\ldots ,n}$ are applied to the *n* rows, then the currents at the *m* columns $z=z_{j=1,\ldots ,m}$ are collected. Exploiting Ohm’s and Kirchhoff’s laws, the expression of the collected currents are as follows:

(1) $$z=G\cdot x=\left[\begin{array}{c} z_{1}=\sum_{j=1}^{n}g_{1,j}x_{j}\\ \vdots \\ z_{m}=\sum_{j=1}^{n}g_{m,j}x_{j} \end{array}\right]$$

which is equivalent to an MVM operation.

The use of arrays of conductive elements for matrix multiplication has been proposed due to the renewed interest in deep learning, where it has been gaining attention as a possible solution to speed up the required computations [6,7]. To maintain the benefits noted above, weight data must be stored in a physical array, with all operations being performed locally. Ideally, the requirements for a memory device to be employed for AIMC are as follows: (i) storage and retention of weights; (ii) a nondestructive readout mechanism; and (iii) the possibility to read and write the entire memory array in a single operation. While (i) and (ii) are conceivable, (iii) is not feasible in conventional memories optimized for random sequential access of size-limited words. Thus, conventional memory elements must be arranged in an array architecture that differs from the architecture of conventional memory, and the employed architecture strongly depends on the type of memory devices being employed.

### 2.1. Memory Devices

A primary class of storage mechanisms in solid-state memories relies on the presence or absence of charge, as seen in Dynamic Random Access Memory (DRAM), Static Random Access Memory (SRAM), and flash memory. An SRAM cell is composed of two CMOS inverters connected in a back-to-back configuration, with charge is confined within barriers formed by FET channels and gate insulators. This configuration provides SRAM with nearly unlimited cycling endurance and sub-nanosecond read and write access times [6,8]. In SRAM, information is stored as electric charge, offering exceptional cycling endurance and rapid access times. On the other hand, a DRAM cell consists of a capacitor that serves as the storage node, which is connected in series with an FET. In flash memory, the storage node is coupled to the gate of an FET. Both SRAM and DRAM support a variety of in-memory logic and arithmetic operations, many of which are based on capacitive charge redistribution, allowing for the storing and sharing of charge across multiple storage nodes. In DRAMs, simultaneous reading of devices along multiple rows enables the execution of basic Boolean functions within the memory array [9,10]. SRAM arrays can also be utilized for matrix-vector multiplication (MVM) operations [11] or designed to perform XNOR operations within each memory cell [12]. For non-binary inputs, capacitors can be used alongside SRAM cells.

Recently, a novel class of memory devices has emerged in which information is stored based on differences in the atomic arrangements of the constituent materials. These differences result in a change in resistance, leading to the designation of these devices as resistive memory devices, or simply memristive devices. Key examples include Phase-Change Memory (PCM), Resistive Random Access Memory (RRAM), and Magnetic Random Access Memory (MRAM). One notable feature of memristive devices is their non-volatile binary storage capability, which facilitates the implementation of logical operations through the interaction between voltage and resistance state variables [5]. Additionally, their ability to store a continuum of conductance values supports the computation of analog matrix-vector multiplications. Memristive devices also exhibit accumulative behavior [13] in which the conductance of devices such as PCM and RRAM progressively increases or decreases with the application of a specific sequence of programming pulses. This non-volatile accumulative behavior can be leveraged in various applications [14].

**Figure 3:**
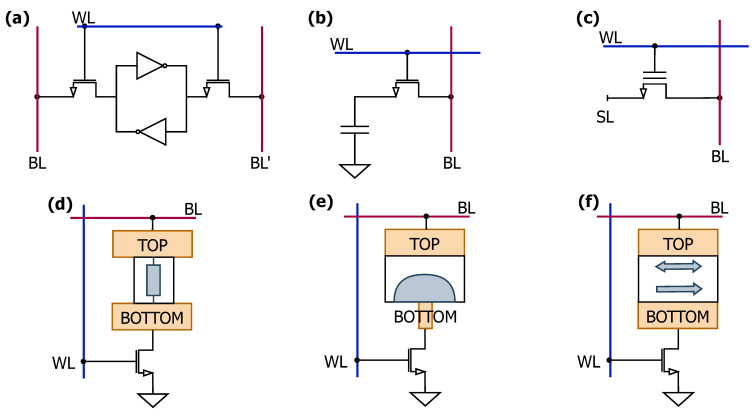
Summary of the most common memory devices employed in IMC systems: (**a**) SRAM, (**b**) DRAM, (**c**) flash memory, (**d**) RRAM, (**e**) PCM, (**f**) MRAM.

In general, one of the main characteristics of a memory device is its access time, which corresponds to the speed with which information can be stored and retrieved. Another key feature is reliability, which refers to the number of times a memory device can be switched from one state to another. A summary of the most common charge-based and resistive memory devices is presented in Figure 3. This review focuses on AIMC systems and applications based on PCM. These technologies are explicated in the following Section.

### 2.2. Phase-Change Memory for Analog In-Memory Computing

PCM operates by exploiting the reversible phase transition of chalcogenide materials between amorphous and crystalline states, allowing for multilevel storage and in-memory computation [15,16]. Advantages include non-volatility, high scalability, and compatibility with existing CMOS technologies. PCM enables fast read/write operations, long-term data retention, and low power consumption, making it suitable for applications in resource-constrained environments [17,18].

Specifically, the possibility of storing information suitable for AIMC contexts has been shown in several works [19,20,21]. This technology allows for the development of fully working AIMC prototypes, as briefly presented in Section 3, as well as related applications discussed in the subsequent sections.

AIMC architectures leveraging PCM have demonstrated remarkable efficiency in MVMs, which represent a fundamental operation in signal processing [22], deep learning [23], and compressed sensing [24,25]. Additionally, as demonstrated in [26], PCM allows for efficient storage and retrieval of a massive quantity of analog values, reducing the need for extensive data movement between memory and processing units.

## 3. PCM-Based AIMC Protoypes

This section presents a comparison of the most recently developed AIMC prototypes exploiting PCM technology.

The prototype presented in [27] consists of an embedded PCM peripheral unit designed to perform signed Multiply-And-Accumulate (MAC) operations directly within a 90-nm CMOS embedded PCM memory with GST cells [28]. Unlike other solutions, this approach does not require modifications to the PCM array, instead leveraging a regulated bitline readout circuit and a drift compensation technique based on conductance ratios. The experimental results demonstrate an MAC accuracy of 95.56%, with performance degradation due to drift remaining below 1% even after 24 h at 85 °C. By integrating these compensation techniques at the peripheral level, the proposed method ensures stable and precise computation over time.

Another work [29] introduced HERMES, a highly optimized PCM-based computing core implemented in 14 nm CMOS technology. This system is designed to support deep learning inference and integrates an array of 256 × 256 PCM cells with dedicated peripheral circuits, including a novel Current-Controlled Oscillator (CCO)-based ADC and a Local Digital Processing Unit (LDPU). The combination of these elements allows the core to achieve high-speed and energy-efficient in-memory computing, supporting fully parallel MAC operations with 8-bit inputs and outputs. When evaluated on machine learning tasks, HERMES achieved 98.3% accuracy on MNIST [30] and 85.6% on CIFAR-10 [31], with an energy efficiency of 10.5 TOPS/W and a performance density of 1.59 TOPS/mm^2^. Compared to other PCM-based AIMC solutions, it offers superior throughput and minimal latency, making it a scalable building block for neuromorphic accelerators.

A third study [32] focuses on one of the main limitations of PCM for AIMC, namely, conductance drift over time, which affects accuracy in long-term operations. To address this issue, the authors proposed a new multilevel programming scheme that improves the stability of PCM cells by gradually crystallizing them from a weak reset state, thereby avoiding abrupt threshold switching. Furthermore, they introduce a differential conductance representation in which each weight is stored as the difference between two conductance values, which inherently compensates for drift. Experimental validation on a 20-kb test chip demonstrated that this technique maintains more than 5-bit precision for matrix vector multiplications even after one day at 180 °C. With a standard deviation of error below 2.2%, this approach significantly enhances the long-term reliability of PCM-based AIMC.

In [33], the authors presented a hybrid SLC-MLC PCM computing-in-memory macro designed for edge devices. This 40 nm 2M-cell prototype supports 8-bit precision and offers an energy efficiency of 20.5 to 65.0 TOPS/W. It employs a hybrid storage scheme utilizing both Single-Level Cells (SLCs) for high signal margin and Multi-Level Cells (MLCs) for area efficiency. Key innovations include a Voltage-Swing Remapping Voltage Sense Amplifier (VSR-VSA) and an Input-Reordering (IN-R) scheme to enhance throughput and energy efficiency. The system achieves a 3.25–15.9 ns access time and demonstrates superior performance in terms of signal margin and energy efficiency compared to previous designs. The hybrid configuration balances accuracy, energy efficiency, and weight density, making it suitable for tiny AI edge applications.

Each of these studies offers a different perspective on improving PCM-based AIMC. The embedded PCM peripheral unit prioritizes computational accuracy without altering memory architecture, making it a practical solution for integration into existing memory systems. In contrast, HERMES focuses on optimizing performance for deep learning workloads, combining high-speed ADCs with efficient digital postprocessing. Meanwhile, the drift compensation technique ensures long-term stability, making PCM viable for applications requiring extended operational reliability. The hybrid SLC-MLC approach provides a balanced tradeoff between signal margin and memory density, optimizing performance for edge computing applications. Table 1 provides a comparative overview of the four approaches.

Further investigations into possible PCM-based AIMC prototypes have also been proposed in [36], where their potential use was assessed in combination with digital multicore clusters for efficient deployment in Deep Neural Network (DNN). The following sections address specific applications of PCM-based AIMC prototypes in the fields of compressive sensing, structural health monitoring, motor control, and in-memory search, which are all common higher-level applications of edge computing.

## 4. Analog PCM-Based Encoder for Compressed Sensing

Compressive Sensing (CS) is a signal processing technique that enables the reconstruction of sparse or compressible signals from a small number of measurements, significantly fewer than required by the Nyquist–Shannon sampling theorem [37,38]. This is achieved by leveraging sparsity, meaning that the signal has a concise representation in some transform domain. The key idea behind CS is that instead of acquiring all signal samples and then compressing them, it is possible to acquire a compressed representation directly through random or structured projections. Reconstruction is then performed using minimization algorithms. CS has applications in medical imaging, wireless communications, and computational photography [39].

**Figure 4:**
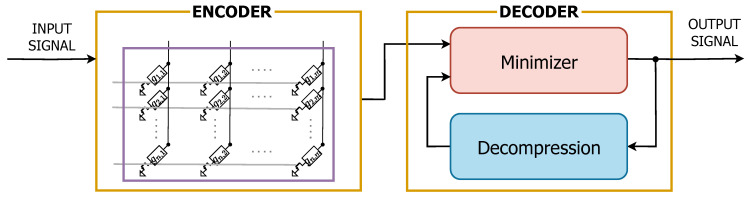
Architecture of a PCM-based system for CS applications with the decoder proposed in [41].

In [40], the authors explored strategies for enhancing the reliability of a PCM-based AIMC encoder for CS applications, as depicted in Figure 4. By addressing the non-idealities inherent in PCM technology, their study aimed to improve data compression efficiency while ensuring robust signal reconstruction. The focus was on both hardware and algorithmic solutions that could mitigate the effects of programming variability and conductance drift, which significantly impact performance over time.

Today, PCM cells still manifest several challenges to be addressed [21]. These devices suffer from programming spread, meaning that the conductance values written into memory can vary unpredictably, as well as from conductance drift. In the latter, values shift over time potentially degrading performance. Because CS relies on an accurate sensing matrix to compress signals before reconstructing them, any uncertainty in the stored values can impact the quality of the final output. Thus, addressing these issues is crucial to making PCM-based AIMC a viable solution for low-power and high-efficiency computing.

### 4.1. AIMC Test Chip and Conductance Models

To evaluate the impact of PCM non-idealities, ref. [34] examined a 90-nm STMicroelectronics prototype designed specifically for AIMC applications. The test chip in their study integrated an embedded PCM (ePCM) unit that stores the MVM coefficients and performs operations in analog form. Instead of relying solely on raw conductance values, their system computes the ratios between stored values, which reduces errors and improves long-term stability.

In addition to the hardware implementation, statistical models can be developed to better understand the behavior of PCM cells. By analyzing experimental data, the extent of programming variability and drift can be quantified over different time intervals. This allows for more accurate simulations of real-world conditions and assessments of different decoding approaches’ performance under these constraints.

### 4.2. Compressed Sensing and Reconstruction Algorithms

Compressed sensing and reconstruction algorithms apply these hardware considerations to the CS framework, where a signal is compressed using a binary sensing matrix and later reconstructed. In this implementation, the nonzero elements of the sensing matrix correspond to PCM cells in a programmed state, while zeroes are represented by cells in their highest resistance state. However, due to the inherent variability in PCM conductance, the actual matrix values may deviate from their ideal targets, introducing uncertainty into the encoding process.

To tackle this challenge, a study by [42] compared different signal reconstruction algorithms. One of the tested approaches was SPGL1 (basis pursuit denoising), which formulates the reconstruction problem as an optimization task with the aim of finding the sparsest signal representation that matches the observed measurements. Another method, Generalized Orthogonal Matching Pursuit (GOMP), takes an iterative approach in which multiple components of the signal are selected at each step to improve efficiency. Finally, Generalized Approximate Message Passing (GAMP) leverages Bayesian inference to estimate the original signal, making it particularly well-suited to handling measurement uncertainties.

### 4.3. Experimental Evaluations and Key Findings

To assess the effectiveness of these approaches, the authors conducted simulations using synthetic signals designed to be sparse in the Discrete Cosine Transform (DCT) domain. They then evaluated how different PCM conductance levels (0.1, 0.4, and 0.7, in normalized units) affected performance, particularly in terms of Reconstruction Signal-to-Noise Ratio (RSNR).

The results revealed important tradeoffs. Higher conductance values tended to improve reconstruction accuracy, as they reduce the relative impact of variability, but this came at the cost of increased power consumption. Among the tested algorithms, GAMP consistently achieved the best reconstruction performance, often exceeding an RSNR of 30 dB when using mid-range conductances. GOMP also performed well, offering a reasonable balance between accuracy and computational efficiency.

Another key finding concerned the effects of conductance drift over time. Without compensation, drift can cause a significant drop in performance. However, by employing the authors’ proposed hardware compensation strategy, their study found that performance degradation can be limited to just 2.3 dB, even under extreme conditions such as 24 h of accelerated aging at 90 °C. This suggests that although drift remains a concern, it can be effectively managed through appropriate design strategies.

The above study highlights the potential of PCM-based AIMC encoders for compressed sensing applications while emphasizing the importance of mitigating programming variability and drift. Their research demonstrates that selecting an appropriate target conductance can balance energy efficiency and reconstruction accuracy, with mid-range conductances (around 0.4) offering the best tradeoff. Furthermore, the implementation of hardware-based drift compensation proves essential for maintaining reliable performance over time.

## 5. PCM in Smart Sensing and Structural Health Monitoring

### 5.1. Background

In recent decades, Structural Health Monitoring (SHM) has become increasingly crucial for ensuring the safety and maintenance of civil infrastructure [43,44]. Many bridges and viaducts built in the last century are now reaching or exceeding their expected lifespan, increasing the risk of structural degradation. The growing demand for traffic on this infrastructure makes it even more urgent to develop advanced systems capable of continuously monitoring structural conditions, detecting potential damage, and preventing catastrophic failures.

While sophisticated vibration-based SHM algorithms have been developed over the years, traditional technologies rely on microcontrollers that require continuous data exchange between the memory and central processing unit [45]. This constant data flow leads to high energy consumption, particularly in distributed monitoring systems where sensors must transmit data wirelessly. Power supply management is a major challenge; frequently replacing batteries is not feasible in large-scale monitoring networks, especially when dealing with vast and numerous structures. To address these limitations, researchers have been increasingly exploring edge computing strategies that allow data processing to occur directly within the sensing nodes, which can significantly reduce transmission needs and overall energy consumption.

In this context, the study presented in [46] explored an innovative approach based on PCM technology, a type of resistive non-volatile memory that enables in-memory computing. The primary goal of this research was to evaluate the effectiveness of PCM for civil infrastructure monitoring, leveraging its ability to process data at the memory level.

**Figure 5:**
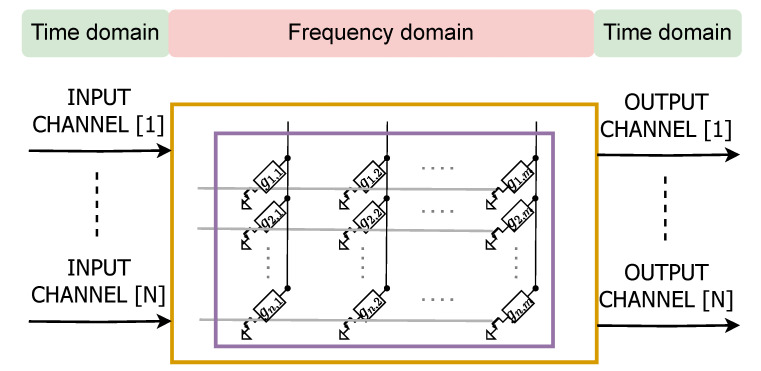
Sketch of the filtering procedure for SHM applications based on PCM devices proposed in [47].

To test this technology, the researchers developed a structural identification algorithm based on one-dimensional convolutional filtering, a mathematical technique that is particularly well suited for analog in-memory computing. The system was implemented on an experimental PCM test unit provided by STMicroelectronics designed with 90-nm CMOS–DMOS technology and a Ge-Sb-Te alloy optimized for automotive applications [28] (see Figure 5). The objective was to extract dynamic and quasi-static structural parameters from vibration data and use them to detect potential structural damage.

### 5.2. Experimental Testing on an Italian Viaduct

To validate the proposed method, the researchers conducted real-world testing on a viaduct of the Italian A24 motorway. Several force–balance accelerometers were installed at various points on the structure to record vibration responses. Data were collected as a 1750 kg vehicle traveled across the viaduct at speeds ranging between 30 and 60 km/h. The goal was to measure the bridge’s dynamic response and evaluate the algorithm’s ability to extract two key indicators of structural health from acceleration data, namely, the mode shapes and curvature profiles.

An important aspect of the study is its comparison of two different filtering approaches:

- Batch filtering, in which signals are processed in a single step using large convolutional filters.
- Recursive filtering, in which data are processed progressively, reducing the number of simultaneous operations.

The results showed that recursive filtering reduced energy consumption by over 90% compared to batch filtering, making it a much more efficient solution for large-scale SHM applications.

In terms of accuracy, the extracted mode shapes closely matched those obtained using the well-established Frequency Domain Decomposition (FDD) technique. This confirmed the effectiveness of the new approach. Although the curvature lines displayed some noise, their overall shape and peak positions were well-defined, demonstrating the system’s capability to correctly detect structural deformations.

Another crucial part of the study was verifying the long-term stability of PCM-based filtering. Because PCM is subject to conductance drift over time, the researchers accelerated the aging process by baking the memory units at 150 °C for 48 h, simulating years of natural degradation. The algorithm continued to perform reliably even after this accelerated aging, proving that PCM can be used for long-term structural monitoring without significant performance loss.

The findings of this study demonstrate that phase change memory technology has the potential to revolutionize sensing devices used for SHM. The proposed method effectively allowed for the identification of key structural parameters while drastically reducing energy consumption, making it a viable solution for large-scale deployment. This research opens the door to new applications of PCM in smart monitoring systems, with the potential to develop ultra-low-power sensors capable of processing data on-site without the need for continuous data transmission.

## 6. PCM for Motor Control

PCM has also been investigated for its role in AIMC-based neural network inference, particularly in motor control applications. Motor control systems increasingly leverage Artificial Intelligence (AI) to process sensor data, optimize actuation, and predict failures [48]. Traditional AI-based control architectures require a microcontroller for real-time control and an additional processor for neural network inference, leading to high latency and energy consumption due to frequent data transfers between memory and processing units. By contrast, AIMC integrates computation directly within non-volatile memory, eliminating the need for continuous data movement and enabling more efficient AI implementations.

### 6.1. Implementation and Methodology

**Figure 6:**
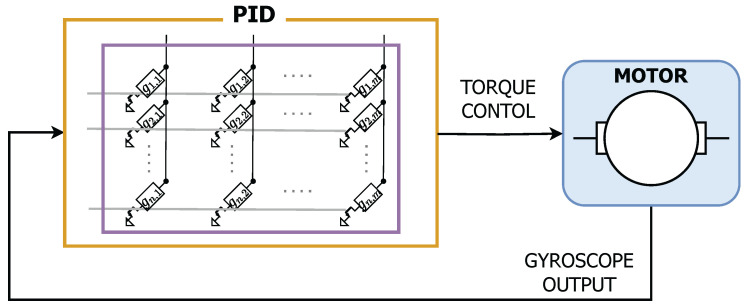
Sketch of the experimental setup for motor control based on PCM devices proposed in [49].

In [49], the authors implemented a simple NN for motor control using an AIMC unit with an embedded PCM array featuring Ge-rich GeSbTe (GST) cells. The NN was designed to regulate the target torque of a brushless three-phase motor using inputs from an accelerometer and a gyroscope. The network consisted of an input layer, a hidden layer, and an output layer, with all weight coefficients and biases mapped onto PCM cells. A sketch of the setup is reported in Figure 6.

The PCM cells were programmed using a SET-staircase (SSC) algorithm that iteratively adjusted the conductance to achieve target values. The NN weights were represented using a binary encoding scheme in which each coefficient was mapped across multiple PCM cells, ensuring a multilevel representation. This approach simplifies programming while maintaining high inference accuracy.

A key innovation of this work is the use of periodic reference conductance ($g_{R}$) calibration to counteract drift effects. Instead of requiring precise tuning of PCM cell conductance, the proposed method adjusts $g_{R}$ dynamically, thereby stabilizing the output voltage of the MAC operations within the AIMC unit.

### 6.2. Experimental Results

The accuracy of NN inference was evaluated under different conditions by measuring the Normalized Root Mean Square Error (NRMSE) across multiple scenarios. The study examined three different conductance levels and evaluated performance over time intervals ranging from one minute to five days.

The results indicated that PCM drift significantly degraded NN accuracy over time in the scenario without compensation, especially at lower conductance levels. The proposed drift compensation technique successfully mitigated this effect, maintaining an accuracy above 96% even after five days. The compensation method proved particularly beneficial at lower conductance levels, ensuring energy-efficient operation without compromising performance.

This study provides experimental validation of an AIMC-based NN for motor control, demonstrating that drift compensation is essential for long-term inference stability. The findings confirm that PCM-based AIMC can achieve high accuracy while reducing power consumption, making it a viable alternative for AI-driven embedded motor control applications.

By addressing the key challenge of conductance drift, this work advances the feasibility of AIMC for real-world AI applications, offering a scalable and energy-efficient solution for future smart motor control systems.

## 7. PCM in Binary Pattern Matching

### 7.1. Background

The research presented in [50] explores the implementation of Binary Pattern Matching (BPM) using an Analog In-Memory Computing (AIMC) unit that incorporates embedded Phase-Change Memory (ePCM). This AIMC unit is designed with a 90-nm CMOS technology by STMicroelectronics and specifically developed to accelerate data-centric computing tasks. The research focused on evaluating the impact of Conductance Time Drift (CTD)—an inherent challenge in PCM-based systems—on pattern recognition accuracy. To mitigate these effects, the study compared two different PCM cell programming algorithms, SET Staircase (SSC) and RESET Staircase (RSC), and analyzed their influence on device performance. The aim of the paper was to characterize and compare the SSC and RSC programming techniques in order to determine their effectiveness in reducing CTD and improving BPM accuracy.

The AIMC unit under investigation performed signed AMC operations in an analog fashion. It consisted of an ePCM array that stores conductance values corresponding to computational weights, while the AIMC circuit executes MAC operations by summing the current contributions of PCM cells. The test vehicle allowed for fine-grained control of programming sequences and drift characterization through an experimental setup that included an evaluation board and Graphical User Interface (GUI). This setup enabled the researchers to program PCM cells with different conductance levels, measure CTD over time, and execute BPM tasks under controlled conditions.

### 7.2. Experimental Testing

Two different multilevel programming approaches were investigated in detail. In the SSC algorithm, a sequence of increasing SET pulses was applied to gradually reach the desired conductance level, while for RSC a sequence of RESET pulses was used to decrease conductance in a stepwise manner. The results showed that SSC provided better control over the programming process, leading to higher success rates in achieving target conductance values. In contrast, RSC exhibited a steeper programming curve, resulting in less precise conductance control and higher sensitivity to drift.

The study evaluated the effects of CTD by programming multiple sets of PCM cells at different conductance levels and monitoring their drift over time. Drift coefficients were extracted using empirical models in order to quantify the rate at which the conductance values degraded. The findings indicated that SSC-programmed cells consistently exhibited lower drift coefficients, making them more stable for long-term computing applications. Additionally, experiments performed under elevated temperature conditions (150 °C annealing) further validated the superior stability of SSC over RSC.

BPM tasks were executed by encoding binary patterns as conductance levels within the ePCM array. The input binary string was compared against all possible stored patterns using MAC operations, with the correct match determined by the highest MAC output. The experiments demonstrated that SSC-programmed PCM cells achieved high BPM accuracy, with the hit rate exceeding 90% in most scenarios. SSC maintained reliable performance even at lower conductance levels, whereas RSC required higher conductance levels to achieve comparable results, making it less efficient in terms of energy consumption.

To further investigate the long-term effects of drift, the study extended BPM analysis through simulations that modeled conductance degradation over time. The simulated experiments included longer binary patterns (up to 9 bits) and additional drift conditions in order to evaluate BPM performance under more challenging scenarios. The results confirmed that SSC remained effective in mitigating drift-induced errors even under prolonged operation and high-temperature stress conditions. These results suggests that SSC represents a more robust programming strategy for PCM-based AIMC applications.

The above research concluded that SSC represents the preferred programming method for BPM tasks in AIMC systems due to its higher success rate in achieving target conductance levels, lower CTD effects, and superior BPM accuracy. These findings have significant implications for the design of PCM-based computing architectures, as they enable more efficient and reliable analog computing operations. This paper provides valuable insights into optimizing AIMC units for future applications where low-power and high-accuracy computing solutions are increasingly in demand, such as AI, machine learning, and large-scale data processing,.

## 8. Using PCM Technology for Human Body Monitoring Applications

PCM devices consume less energy compared to traditional memory types such as DRAM and SRAM, making them particularly useful for wearable monitoring devices that require long battery life [17,51]. PCM offers faster access times compared to flash memory, allowing for near real-time data processing, which is crucial for continuous monitoring of vital parameters. Additionally, PCM has greater resistance to write/read cycles compared to flash memory, making it ideal for applications such as human body monitoring that require frequent data updates [52].

As far as the most common body monitoring applications are concerned, AIMC systems based on PCM can analyze ECG data in cardiac monitoring in real time, helping to detect anomalies such as arrhythmias or heart attacks with high precision and timeliness. For diabetic patients, wearable devices can continuously monitor blood glucose levels, using AIMC algorithms to predict and prevent hypoglycemic or hyperglycemic episodes. In sleep monitoring, wearable sensors can collect sleep data such as movements and heart rate, then use AIMC to identify sleep disorders such as sleep apnea.

## 9. Conclusions

The use of Phase-Change Memory (PCM) in edge computing and Analog In-Memory Computing (AIMC) represents a groundbreaking opportunity to enhance energy efficiency, processing speed, and hardware integration. The applications analyzed in this work, summarized in Table 2, demonstrate how PCM can transform computational paradigms by reducing data movement between memory and processing units along with its use in applications such as encoding for compressed sensing, structural health monitoring, neural network acceleration, and binary pattern matching.

Looking ahead, future directions in PCM-based in-memory computing include the development of more reliable and efficient cell architectures, improvements in material composition to enhance switching speed and endurance, and advanced error correction and calibration techniques to handle variability [53]. Integration with 3D stacking and heterogeneous computing platforms is essential in order to maximize performance and density. On the algorithmic side, there is growing interest in designing machine learning models and software stacks that are tailored specifically for the characteristics of in-memory computing hardware [54]. Additionally, research into hybrid systems that combine PCM with other emerging memory technologies such as ReRAM or MRAM could unlock new capabilities and optimize performance across a range of tasks [55]. As these developments mature, PCM-based in-memory computing holds the potential to revolutionize data processing, offering an efficient, scalable, and high-performance alternative to conventional computing paradigms [18].

For further analysis, Table 3 summarizes the typical parameter counts in terms of order of magnitude for commonly used neural network models across various application domains. These values are crucial when evaluating the feasibility of deploying such models on PCM-based AIMC systems, where hardware limitations in terms of array size, precision, and energy efficiency must be carefully considered.

Current PCM-based AIMC experimental prototypes can support networks with up to $10^{5}$ or $10^{6}$ synaptic weights, which aligns well with small-scale models such as MLPs and CNNs trained on datasets such as MNIST. These models have been implemented successfully in hardware, achieving competitive energy efficiency and throughput [54,56,57]. For mid-sized networks such as ResNet-18 or AlexNet, which typically involve $10^{7}$ to $10^{8}$ parameters, AIMC faces scalability challenges due to device variability, noise, and retention issues inherent in analog devices. While these models are still within reach for advanced research prototypes, they generally require architectural partitioning or tiling strategies, and often rely on hybrid digital–analog systems for control and precision correction [58]. When scaling up to transformer-based models such as BERT or GPT-2 with over $10^{8}$ parameters, the limitations of analog hardware become more pronounced; PCM arrays lack the precision and density to efficiently handle such large models without significant compromises in accuracy, necessitating the use of quantization, pruning, or offloading strategies. For very large models such as GPT-3 or image generation architectures such as Stable Diffusion, which involve up to $10^{11}$ parameters, AIMC is currently impractical as a full solution. Even in hybrid systems, these models require computing resources far beyond what current analog memory arrays can support. In these cases, AIMC is being explored as a domain-specific accelerator for selected layers rather than as an end-to-end platform. Overall, while AIMC with PCM is promising for energy-efficient inference in small and moderately sized networks, substantial progress in array design, fabrication, and algorithmic robustness is required to enable deployment at the scale of state-of-the-art deep learning models.

To conclude, despite existing challenges such as conductance drift over time, proposed PCM-based AIMC solutions such as compensation techniques and novel circuit architectures confirm the potential for widespread adoption in real-world applications. Future integration with advances in materials and computational models could further solidify the role of PCM technology in the emerging technological landscape. The employment of AIMC systems based on PCM for human body monitoring offers promising advantages in terms of energy efficiency, speed, and reliability. With further developments and cost reductions, these systems have the potential to revolutionize health monitoring and significantly improve patients’ quality of life.

## Figures and Tables

**Table 1:** Comparison between the most recent PCM-based AIMC prototypes.

Feature	[34] (90 nm)	[35] (14 nm)	[32] (90 nm)	[33] (40 nm)
**Focus**	AIMC unit for MAC operations	High-speed PCM-based DL inference	PCM drift compensation in MLC	Hybrid SLC-MLC PCM for edge devices
**Core innovation**	Bitline readout + conductance ratio for drift correction	Linearized CCO-based ADCs + local digital processing	Differential conductance representation for drift immunity	Hybrid SLC-MLC storage + VSR-VSA + IN-R scheme
**Accuracy**	95.56% MAC accuracy	98.3% (MNIST), 85.6% (CIFAR-10)	5-bit precision after 1 day at 180 °C	0.81% degradation (CIFAR-100)
**Energy efficiency**	Not specified	10.5 TOPS/W	Not specified	20.5–65.0 TOPS/W
**Performance under drift**	<1% degradation after 24 h at 85 °C	High-speed operation with DL inference	σ<2.2% error after extended drift	Enhanced signal margin and throughput

**Table 2:** Comparison of PCM AIMC across different applications.

Application	Topic	Main Challenges	Proposed Solutions	Key Results
**Compressed Sensing**	Develop an AIMC encoder to reduce data needed for signal reconstruction	Conductance drift, programming variability	Drift compensation techniques and conductance level optimization	Reduced reconstruction error, better balance between energy consumption and accuracy
**Structural Health Monitoring**	Reduce energy consumption in structural health monitoring systems	High energy consumption of traditional systems, PCM memory instability	Convolutional filtering with PCM, reduced data transmission	90% energy savings, high reliability even after accelerated aging
**Motor Control**	Improve the efficiency of neural networks for embedded applications	Conductance drift, precision loss over time	Periodic calibration of reference conductance	Accuracy above 96% after 5 days of operation
**Binary Pattern Matching**	Develop an efficient AIMC system for binary pattern matching	Conductance drift, programming variability	SSC programming for greater stability	Over 90% accuracy in pattern recognition

**Table 3:** Orders of magnitude of parameters in common neural models.

Category	Model	Parameters (Order of Magnitude)
Small networks (MNIST)	MLP, CNN	∼$10^{5}$–$10^{6}$
Image classification	ResNet, VGG, AlexNet	∼$10^{7}$–$10^{8}$
Natural language processing (NLP)	BERT, GPT	∼$10^{8}$–$10^{9}$
Advanced generation	GPT-3, Stable Diffusion	∼$10^{9}$–$10^{11}$
